# Multiparametric CT-Based Adult Age Estimation Using Thoracic Vertebral Degenerative Changes, Vertebral Bone and Paraspinal Muscle Attenuation: A Regression-Based Approach

**DOI:** 10.3390/diagnostics16162528

**Published:** 2026-08-11

**Authors:** Emre Nuri Igde, Adalet Elcin Yildiz, Ramazan Akcan, Aysun Balseven Odabasi

**Affiliations:** 1 The Council of Forensic Medicine, Ankara Group Chairmanship, Ankara 06300, Türkiye; 2 Department of Radiology, Faculty of Medicine, Hacettepe University, Ankara 06230, Türkiye; aelcinyildiz@hacettepe.edu.tr; 3 Department of Forensic Medicine, Faculty of Medicine, Hacettepe University, Ankara 06230, Türkiye; ramazan.akcan@hacettepe.edu.tr (R.A.); balseven@hacettepe.edu.tr (A.B.O.)

**Keywords:** forensic medicine, forensic anthropology, age estimation, computed tomography, osteophytes, attenuation, regression analysis

## Abstract

**Background/Objectives**: Adult age estimation remains challenging because degenerative processes lack uniform progression and show substantial interindividual variability. This study aimed to evaluate the association between thoracic vertebral degeneration and chronological age using multidetector computed tomography (MDCT) and to develop regression-based age estimation models. **Methods**: A total of 240 individuals (120 females and 120 males) were included. Four parameters—osteophyte formation, facet joint degeneration, vertebral corpus bone attenuation, and paraspinal muscle attenuation—were assessed, generating 15 derived variables used for regression models. Univariable and multivariable linear regression analyses were conducted in combined and sex-stratified samples. Internal validation was conducted using 1000 bootstrap resamples. **Results**: Among single variables, linear osteophyte measurement (L0) showed the strongest association with age in the combined sample (R^2^ = 0.682), with a root mean squared error under 10 years. Two multivariable regression models were constructed. In the combined development sample, model 1, which allowed both morphological and attenuation-based variables to enter the selection procedure, achieved an R^2^ of 0.84 with a root mean squared error of 6.93 years. Morphology-based model 2 yielded an R^2^ of 0.75 and a root mean squared error of 8.58 years. Bootstrap internal validation showed modestly lower performance than the apparent estimates. **Conclusions**: Thoracic vertebral degenerative and attenuation-based parameters assessed by MDCT demonstrate potential as a complementary method for adult age estimation. The proposed dual-model strategy may offer flexibility for evaluating thoracic vertebral parameters under different forensic conditions. External validation in multicenter and postmortem cohorts is needed to establish the generalizability of the models and assess their suitability for routine forensic application.

## 1. Introduction

Age estimation, along with sex estimation, represents a fundamental component of biological profile reconstruction in forensic anthropology and forensic medicine, particularly in situations involving unidentified decedents or skeletal remains [1]. While age estimation in subadults can be performed with relatively higher reliability through assessments of bone and dental maturation, estimating age in adults is considerably more challenging, as it utilizes degenerative processes that demonstrate a lack of uniform progression and considerable interindividual variability [2,3].

In adults, articular degenerative changes have been suggested as potential indicators of estimation of age-at-death [3]. Established methods focusing on the pubic symphysis [4], auricular surface [5], and sternal end of ribs [6] demonstrate age-related morphological progression; still, their reported accuracy varies considerably across studies and different populations. Differences in scoring systems, interobserver variability, and inconsistent error ranges limit reproducibility, highlighting the necessity for population-specific and statistically robust models [7].

Multiple studies have reported associations between age and osteophyte formation in the vertebrae, which have attracted increasing attention in this area [8,9,10]. Nonetheless, those studies have analyzed osteophytes as an individual morphological parameter. Facet joint osteoarthritis has been infrequently included in models [11], and quantitative parameters such as vertebral bone and paraspinal muscle attenuation are still inadequately represented in multivariable models. This represents an important gap in forensic adult age estimation, as ageing is a multidimensional process rather than a single morphological phenomenon. In addition to morphological degenerative changes, including osteophyte formation and facet degeneration, age-related decreases in vertebral trabecular attenuation [12,13] and fatty infiltration of the paraspinal muscles [14,15], reflected by reduced muscle attenuation on CT, may provide complementary quantitative information.

The cervical, thoracic, and lumbar regions of the vertebral column differ in their mobility and loading characteristics. The cervical spine contributes substantially to neck movement [16], whereas the lumbar spine is subjected to compressive loading [17]. By contrast, the thoracic spine has relatively limited mobility because of the stabilizing effect of the rib cage [16]. This biomechanical characteristic provides a rationale for investigating age-related degenerative changes in the thoracic region. In addition, the thoracic spine is frequently included in routine clinical CT examinations, which facilitates the availability of imaging data for retrospective assessment of age-related changes.

Despite increasing interest in vertebral degeneration as an indicator of adult age-at-death, the majority of current models are largely based on morphological scoring systems [8,9,11]. There is a lack of attempts to merge different aspects of degeneration into a regression model, and population-specific models based on CT scans are rare. Against this background, the integration of visually assessed degenerative traits with objective attenuation parameters could offer a holistic biological-statistical perspective.

Accordingly, this study had two primary objectives: (1) to evaluate the association between thoracic vertebral degenerative changes and chronological age in an adult population using thoracic multidetector computed tomography (MDCT) of living individuals, and (2) to develop and internally validated regression-based models for age estimation.

## 2. Materials and Methods

### 2.1. Study Design and Sample

This retrospective cross-sectional study evaluated thoracic MDCT scans performed at the Department of Radiology, Hacettepe University Hospital, between 1 January 2022 and 16 May 2022. Clinical indications for thoracic CT and available medical records were reviewed using the hospital information system (NUCLEUS Hospital Information Management System). Exclusion criteria were applied to eliminate conditions that could potentially affect vertebral morphology or influence attenuation-based measurements. Cases demonstrating vertebral pathological lesions, prior vertebral trauma or surgery, congenital spinal anomalies, pelvic or lower extremity deformities, neurological disorders (including cerebrovascular events or neuropathy), lipodystrophy, neoplastic disease, rheumatologic disease, immunodeficiency, or prolonged corticosteroid use were excluded from the study. Individuals with documented ICD-coded diagnoses of obesity or osteoporosis were also excluded because of their potential effects on paraspinal muscle and vertebral attenuation. After application of the exclusion criteria, eligible cases were stratified by single-year age and sex. From each age–sex stratum, two individuals were randomly selected, resulting in a balanced sample of 240 individuals, with four individuals for each single-year age from 20 to 79 years (Table S1).

Ethical approval was obtained from the Hacettepe University Non-Interventional Clinical Research Ethics Committee (Approval No: 2022/11-52; Date: 21 June 2022).

### 2.2. Data Acquisition

Thoracic CT images (contrast-enhanced and non-contrast) were obtained with 16-slice (Somatom Sensation, Siemens AG, Erlangen, Germany; Light Speed, GE Healthcare, Chicago, IL, USA; Optima CT540, GE Healthcare, Chicago, IL, USA) or 64-slice (Somatom Perspective, Siemens Healthineers, Erlangen, Germany) multidetector CT scanners. Scanning parameters were as follows: tube voltage 100–120 kV, tube current 50–120 mAs, matrix 512 × 512, field of view 350 × 350 mm, and slice thickness of ≤2 mm. MDCT datasets were retrieved from the institutional PACS (Picture Archiving and Communication Systems) workstation and reconstructed to obtain MPR (multiplanar reformations). Axial source images were reconstructed with a slice thickness of 1 mm and subsequently reformatted into coronal and sagittal planes. Morphological assessment of the thoracic vertebrae was performed using bone kernel reconstruction. Soft tissue kernel reconstructions were additionally utilized for quantitative muscle attenuation measurements.

### 2.3. Parameters and Variables

Four primary parameters were evaluated: osteophyte formation, facet joint degeneration, vertebral corpus bone attenuation, and paraspinal muscle attenuation. Osteophyte formation and facet joint degeneration were assessed as morphology-based degenerative parameters. Vertebral bone attenuation and paraspinal muscle attenuation were included as quantitative CT-derived parameters. Previous studies have reported associations between vertebral CT attenuation and bone density [18], suggesting that attenuation values may provide an indirect estimate of bone mineral density. In the present study, vertebral bone and paraspinal muscle measurements were based on CT attenuation values expressed in Hounsfield units (HU).

Osteophyte formation was assessed using both linear measurements and morphological grading according to a modified version of the method described by Chiba [8] (Table 1). All thoracic vertebrae were assessed. For each vertebral body, both superior and inferior margins were examined separately on axial images. Accordingly, a total of 24 osteophyte sites were assessed across the thoracic spine. For linear classification, osteophyte length was defined as the maximal distance between the vertebral body margin and the tip of the osteophyte and was categorized into six stages according to predefined thresholds. Morphological assessment was performed on sagittal and coronal reformatted images, and the presence and extent of spur formation or bridging between adjacent vertebral bodies were graded using a four-stage classification.

Facet joint degeneration was graded using a modified version of the Weishaupt classification system [19]. Although the original grading system was developed for the lumbar facet joints, the thoracic facet joint spaces appeared narrower in our dataset. The grading criteria were therefore adapted to account for the anatomical characteristics of the thoracic region (Table 1). Both facet joints were evaluated at each vertebral level, and the higher degenerative grade was recorded.

Vertebral bone and paraspinal muscle attenuation were quantified in Hounsfield units. To provide regional representation of the thoracic spine, the vertebrae were divided into upper (T1–T4), middle (T5–T8), and lower (T9–T12) segments. T4, T8, and T12 were selected as representative levels for these three regions. Bone attenuation was measured on axial images with HU. Measurements were obtained by placing a manually defined region of interest within the mid-vertebral corpus, avoiding cortical bone and focal lesions.

Paraspinal muscle attenuation was measured at T4, T8, and T12 using axial images obtained at the midpoint of each vertebral body. Manually defined regions of interest were placed bilaterally within the multifidus and erector spinae muscles. Attenuation values were recorded in HU for each side, and bilateral mean values were calculated at each vertebral level.

**Figure 1:**
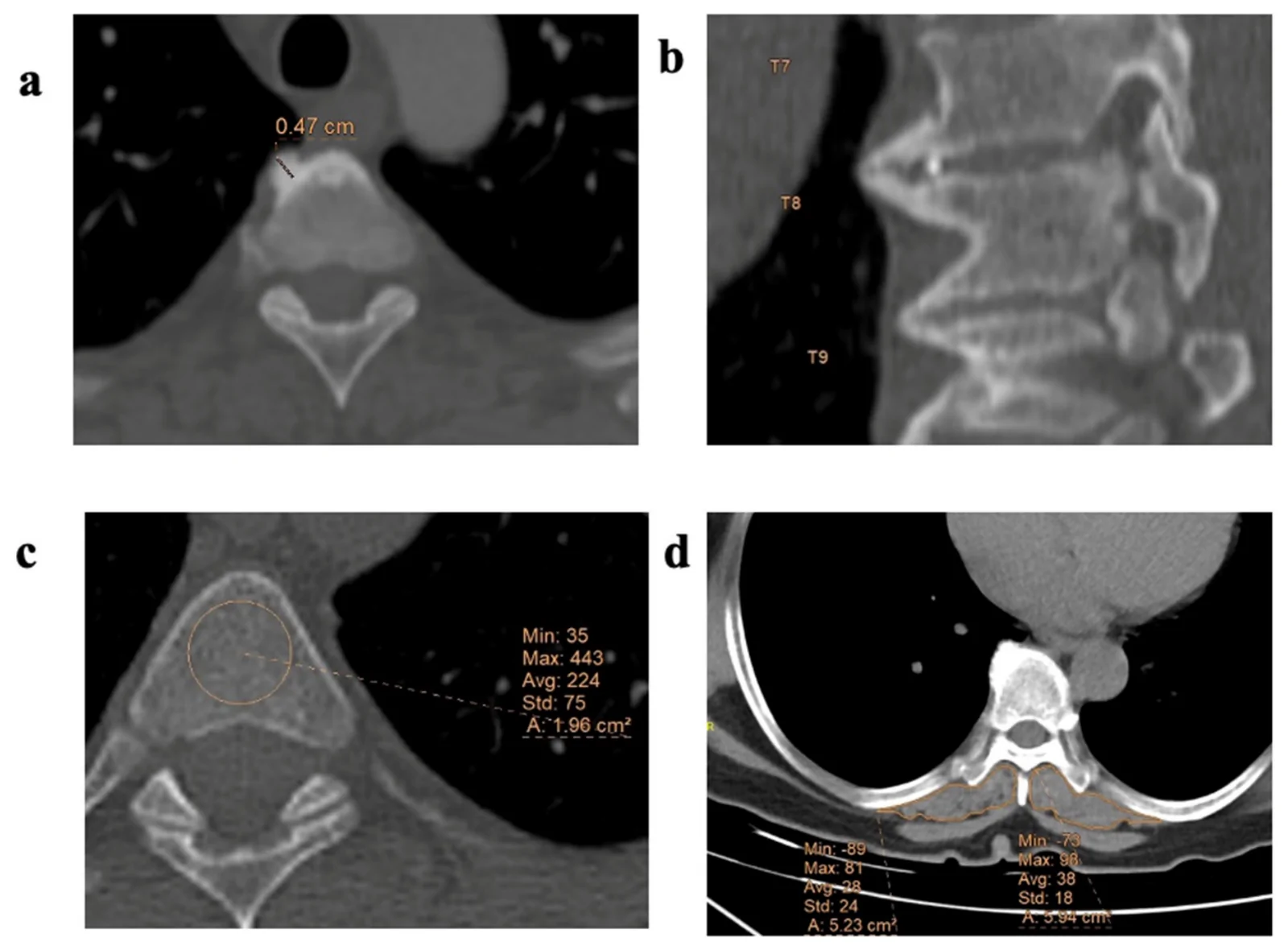
Representative CT images demonstrate the measurement parameters used in the study. (**a**) grade 3 according to the linear osteophyte classification; (**b**) grade 3 in the morphological osteophyte classification at superior and inferior end plate of T8 vertebra; (**c**) vertebral corpus bone attenuation measurement at the T8 level; (**d**) paraspinal muscle attenuation measurement at the T8 level.

**Figure 2:**
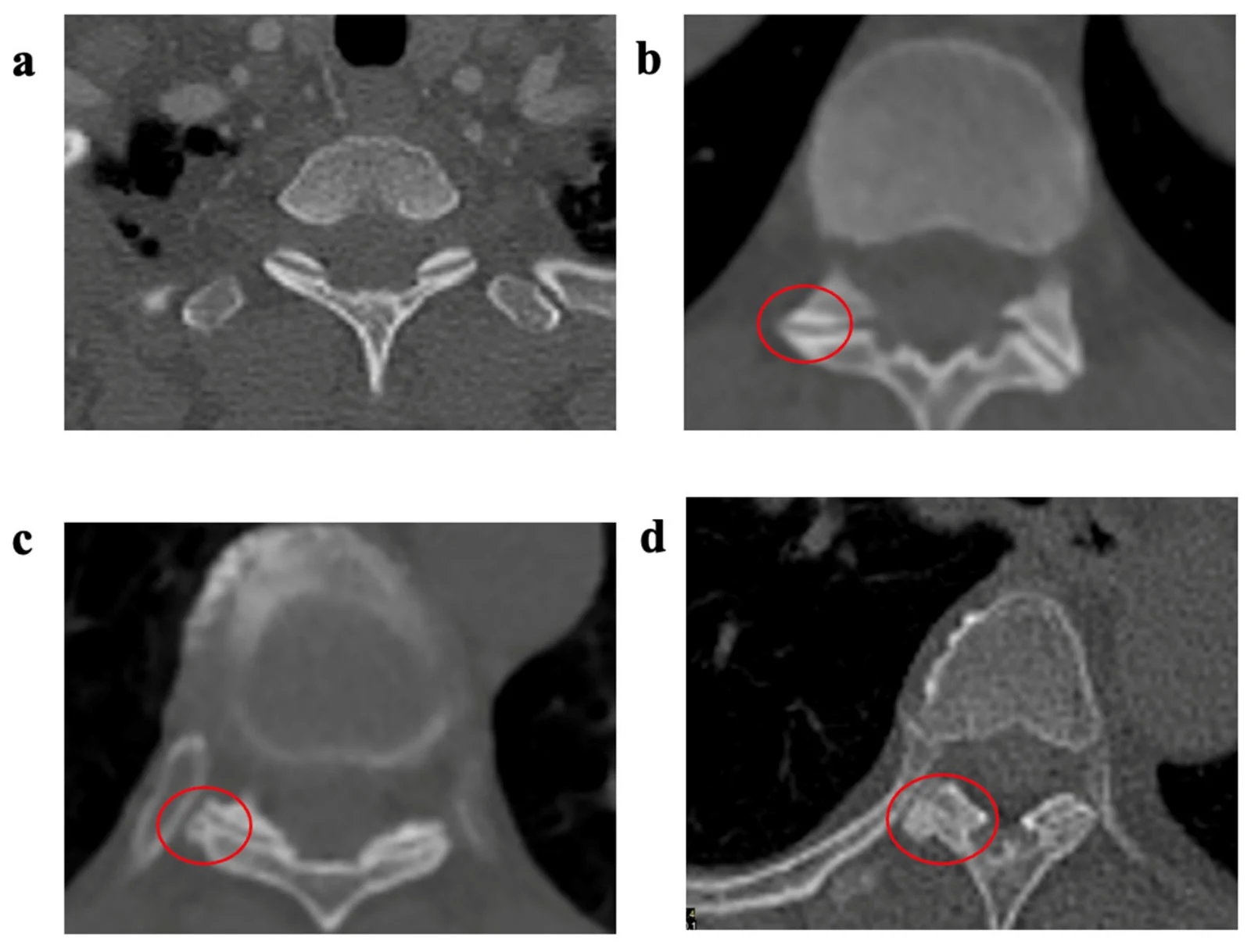
Representative axial CT images illustrating the grading of thoracic facet joint degeneration. (**a**) Grade 0, showing normal bilateral facet joints. (**b**) Grade 1, showing a small marginal osteophyte. (**c**) Grade 2, showing moderate osteophyte formation. (**d**) Grade 3, showing advanced degeneration with facet joint fusion. The relevant degenerative findings are indicated by circles in panels (**b**–**d**).

A total of 15 variables were generated from the four primary parameters described above. Definitions of variables and scoring are provided in Table 2 and Figure 1 and Figure 2.

### 2.4. Statistical Analysis

Descriptive statistics were calculated for all variables. Normality was assessed using the Shapiro–Wilk test. Sex-based comparisons were performed using the independent-samples t-test or the Mann–Whitney U test, as appropriate for the distribution of the data. Differences across 10-year age groups were assessed using one-way analysis of variance (ANOVA) or the Kruskal–Wallis test, according to the distributional characteristics of each variable. Correlations between chronological age and variables were assessed using Spearman’s rank correlation coefficient.

Univariable linear regression analyses were conducted separately for males, females, and the combined sample to evaluate the individual predictive value of each variable. Chronological age was treated as a continuous dependent variable; therefore, regression-based modelling was selected to develop age-estimation models.

In this study, morphology-based degenerative parameters refer to visually graded degenerative changes in the vertebrae, including osteophyte formation and facet joint degeneration, independent of quantitative attenuation measurements. Two regression-based models were developed. Model 1 was non-restricted and included all eligible variables, whereas model 2 was restricted to morphology-based degenerative parameters, including osteophyte formation and facet joint degeneration. Multivariable linear regression models were constructed separately for males, females, and the combined sample using a stepwise method. Multicollinearity among the retained predictors was assessed using variance inflation factors (VIFs), with values below 5 considered acceptable.

Regression assumptions were examined using residual diagnostics. In the final models, the residuals did not show a significant deviation from normality. Although L-max is an ordinal variable, it was entered as a continuous predictor in the primary regression analyses. As a sensitivity analysis, L-max was additionally modelled as a categorical variable to assess whether this coding assumption affected model performance. Because categorical modelling did not significantly improve model fit, the continuous specification was retained in the final models.

Model performance was assessed using coefficients of determination (R^2^), root mean squared error (RMSE), and MAE. Predictive performance was further evaluated by calculating absolute error distributions (in years) and the proportion of estimates within predefined error intervals. In addition, decade-specific performance was analyzed by stratifying the sample into 10-year age groups and calculating accuracy and bias for each decade. Bias was defined as observed age minus predicted age; positive values therefore indicated age underestimation, whereas negative values indicated overestimation.

Internal validation was conducted using 1000 bootstrap resamples, with the stepwise variable-selection procedure repeated within each resample. Optimism-corrected estimates of R^2^, RMSE, and MAE were calculated. Out-of-bag predictions were also examined to assess prediction error and bias in observations not included in the corresponding bootstrap samples.

For each individual, a 95% prediction interval was calculated from the fitted regression model.

Intra- and inter-observer reliability was assessed using the intraclass correlation coefficient (ICC). Thirty cases were selected using stratified random sampling across age decades and sex to ensure representation throughout the study age range. The initial evaluation was performed by one researcher, who reevaluated the selected cases one month later for intra-observer reliability. The same cases were independently evaluated by a second researcher for inter-observer reliability. The cases were anonymized, and age and sex information were concealed from the researchers during the assessments. Reliability was evaluated for all study parameters, and ICC values were reported with 95% confidence intervals. ICC values were interpreted as follows: <0.50 poor reliability; 0.50–0.75 moderate; 0.75–0.90 good; and >0.90 excellent reliability [20]. Statistical analyses were performed using SPSS software version 27.0.1. (IBM Corp., Armonk, NY, USA) and R version 4.5.3 (R Foundation for Statistical Computing, Vienna, Austria). Bootstrap internal validation was conducted using the boot package. A *p*-value <0.05 was considered statistically significant.

## 3. Results

Intra-observer reliability was excellent across all evaluated variables, with ICC values ranging from 0.925 to 0.991. Inter-observer reliability ranged from good to excellent, with ICC values from 0.835 to 0.985. The highest intra-observer agreement was observed for L-sum (ICC = 0.991, 95% CI: 0.982–0.996), and the highest inter-observer agreement for M3 (ICC = 0.985, 95% CI: 0.970–0.993). Facet joint variables showed the lowest inter-observer agreement. For F0, which was retained in the final regression models, the intra-observer ICC was 0.967 (95% CI: 0.931–0.984) and the inter-observer ICC was 0.835 (95% CI: 0.270–0.943). Although the point estimate for inter-observer reliability of F0 indicated good agreement, its wide 95% confidence interval indicated considerable uncertainty (Table S2).

Descriptive statistics for all variables stratified by sex are presented in Table 3. The degree and number of osteophyte formation and vertebral bridging were slightly higher in males; however, statistically significant sex differences were observed only for L-max, L2–L5, and M3 parameters. In contrast, facet joint degeneration scores were higher in females (*p* < 0.05). With regard to bone and muscle attenuation measurements, no significant sex differences were observed, except for paraspinal muscle attenuation at the T12 level, which differed significantly between males and females (*p* < 0.01).

Depending on data distribution, one-way ANOVA or Kruskal–Wallis tests were applied. All variables exhibited statistically significant age-related differences (*p* < 0.001). These findings showed that osteophyte formation and facet joint degeneration increased with age, whereas vertebral bone and paraspinal muscle attenuation decreased. Correlation analyses supported these age-related patterns, with all evaluated parameters showing significant associations with chronological age (*p* < 0.001) (Table S3).

Table S3 presents the univariable regression equations together with the corresponding R^2^, RMSE, and MAE values. Osteophyte-related variables generally showed higher coefficients of determination and lower error estimates than attenuation-based variables. Among the attenuation measures, variables obtained at T12 had the greatest explanatory power. In females, F0 and L0 showed the strongest univariable associations with age, each yielding an R^2^ of 0.655 and greater explanatory capacity than the remaining variables. The RMSE for these models was 10.17 years. In males, L0 also emerged as a significant predictor (R^2^ = 0.714, RMSE = 9.26). Similarly, L0 exhibited the strongest univariable associations with age in combined sample (R^2^ = 0.682), with the RMSE remaining below 10 years.

Two multivariable models were constructed: model 1 included all eligible variables (non-restricted model), whereas model 2 was based solely on morphology-based parameters (osteophyte and facet joint degeneration). In the combined sample, model 1 retained L0, F0, L-max, B-T12, and M-T12, whereas model 2 retained L0, F0, and L-max (Table 4). In the combined development sample, the apparent performance of model 1 yielded an R^2^ of 0.84, a RMSE of 6.93 years, and a MAE of 5.53 years, while model 2 achieved an R^2^ of 0.75 with a RMSE of 8.58 years and a MAE of 6.91 years in the combined sample. Bootstrap internal validation showed a modest decrease in model performance (Table 5). Sex-specific models and performance estimates are provided in Table 4 and Table 5.

**Figure 3:**
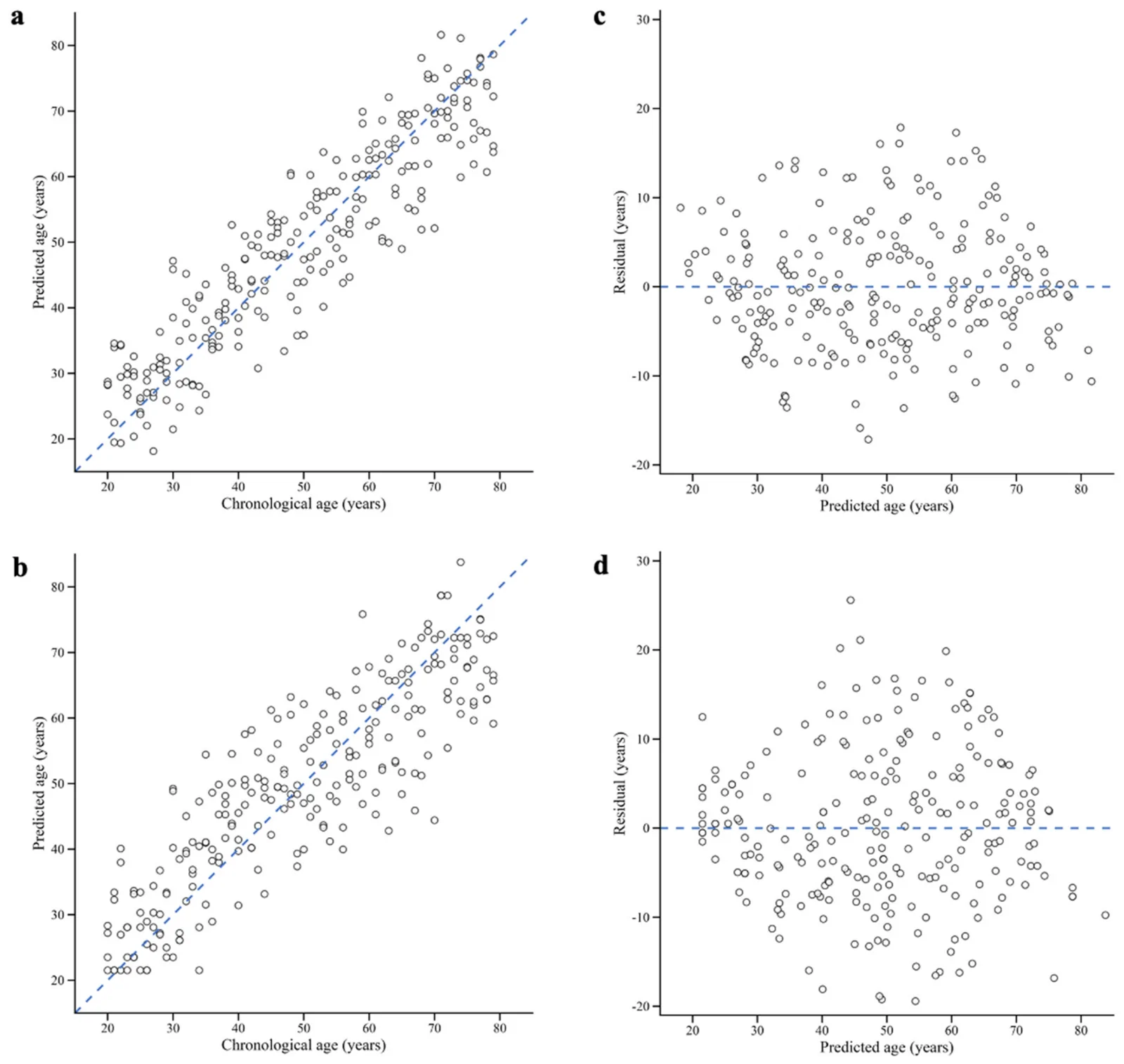
Agreement between observed and predicted age in multivariable regression models. Scatter plots showing the relationship between observed and predicted age values for the two multivariable regression models. (**a**) Model 1, (**b**) Model 2; the diagonal line represents perfect agreement. Distribution of residuals is homogeneous for model 1 (**c**), while model 2 does not show regular residuals distribution (**d**). The horizontal line represents zero residual.

Prediction within ±10 years of chronological age was achieved in 84.3% of cases for model 1 and in 75.0% for model 2 in the combined development sample (Table 6). Decade-specific analysis showed that model 1 had lower MAE values than model 2 across most age groups in the combined and sex-specific samples. In the combined sample, model 1 showed relatively limited variation in MAE across decades, ranging from 4.90 to 5.95 years, whereas the MAE of model 2 increased from 4.85 years in the 20–29-year group to 8.32 years in the 70–79-year group. Decade-specific analysis showed an age-related pattern of prediction bias for model 2, with age overestimation in individuals in their 30s and underestimation in those in their 70s (Table 7). Figure 3 illustrates the predictive performance of both models in the combined sample. Out-of-bag estimates were less favourable than the corresponding apparent estimates. The corresponding out-of-bag error distributions and decade-specific results are provided in the Supplementary Tables S4 and S5. The mean width of the 95% prediction interval was 28.01 years for model 1 and 34.38 years for model 2. Within the development sample, observed age was within the corresponding 95% prediction interval in 95.4% of cases for model 1 and 96.7% for model 2.

## 4. Discussion

This study examined the potential use of thoracic vertebral degenerative changes and attenuation-related parameters obtained from CT imageing for adult age estimation. The results indicate that both visually graded degenerative features and attenuation measurements are associated with chronological age and can be incorporated into regression-based prediction models. Among the evaluated variables, those describing the osteophyte-related variables showed the highest predictive value. The addition of further variables was associated with better apparent model performance. Overall, the results indicate that vertebral degenerative changes may serve as biological indicators of aging and support the use of CT-derived features as complementary information in adult age estimation.

Previous studies of adult age estimation have largely assessed osteophytes through visual grading systems or CT-based morphometric measurements. Several grading systems have been proposed, including those described by Snodgrass, Watanabe, and Chiba [8,9,21]. In present study, Chiba scoring system [8] was selected because of its reported reproducibility and practical suitability for assessment. Facet joint degeneration was graded using a modified Weishaupt classification [19], and vertebral bone and paraspinal muscle attenuation were measured in Hounsfield units. Reliability analysis showed excellent intra-observer agreement and good-to-excellent inter-observer agreement across the evaluated variables, supporting the reproducibility of the assessment protocol. However, the wide confidence interval for the inter-observer ICC of F0 indicates limited precision in the reliability estimate for this variable.

In the present study, particular emphasis was placed on the thoracic spine because of its relative anatomical stability conferred by the rib cage [16] and its relatively lower axial loading compared with the lumbar region. Although previous studies have yielded some heterogeneous results, Snodgrass and Praneatpolgrang demonstrated relatively high predictive performance in models derived from thoracic vertebrae [22]. These findings provide a rationale for investigating the thoracic spine as a potential region for adult age estimation; however, they should not be interpreted as evidence of demonstrated advantage over other spinal regions.

Sex-related differences in osteophyte formation have been reported [23], although the underlying mechanisms remain unclear and may involve differences in biomechanical loading patterns, stature, occupational exposure, and hormonal factors. Although some studies reported largely similar degenerative patterns between males and females [8,21], our findings demonstrated a higher degree and number of osteophyte formations (L-max, L2–5, M3) in males, whereas some variable (F2-3) related to facet degeneration was higher in females. Sex-related differences in the attenuation variables were limited. Differences in the variables retained in the sex-specific models may partly reflect these sex-related patterns, although this interpretation remains exploratory.

In earlier regression-based approaches, total osteophyte scores were commonly used as predictors of chronological age [11,24]. Chiba et al. expanded this strategy by incorporating additional variables, such as the number of vertebrae with clearly developed osteophytes and the maximum osteophyte grade, thereby improving predictive performance of model [8]. Studies based solely on total osteophyte scores have reported minimum errors of approximately 10.37 years (standard error of estimate, SEE) in the overall sample [24]. In Chiba’s regression model, the number of vertebrae demonstrating clear osteophyte formation emerged as the most influential predictor for both males (R^2^ = 0.796, SEE = 9.88) and females (R^2^ = 0.802, SEE = 9.55) [8]. This parameter is conceptually comparable to the L2–5 variable used in the present study, which represents the cumulative number of vertebral levels with advanced osteophyte formation above a predefined severity threshold. In the combined sample, L0 emerged as the strongest individual predictor of age (R^2^ = 0.682, RMSE = 9.75) and showed a similar pattern of performance in the sex-specific analyses. Among females, the F0 variable yielded a similar level of predictive accuracy (R^2^= 0.655, RMSE = 10.17). Overall, the predictive performance of the present models appears broadly comparable to previously published findings.

In the course of regression modelling, attenuation-related variables corresponding to T12 level demonstrated relatively higher coefficients of determination compared with other vertebral levels. The stronger associations observed at T12 may reflect the distinctive biomechanical structure of the thoracolumbar junction, a region characterized by changes in load transmission and structural adaptation [25]. Regional differences in bone attenuation and degenerative change have also been reported within the thoracic spine, with the lower thoracic levels appearing more affected than the upper segments [26]. These factors may help explain the findings at T12, although this interpretation remains speculative because regional loading was not measured directly.

A multivariable approach allows several age-related features to be considered simultaneously and may therefore provide advantages over models based on a single predictor. Using only osteophyte-related variables, Schanandore et al. obtained one of the highest reported coefficients of determination in this area [27]. Their findings support the potential benefit of combining multiple degenerative indicators within a single regression model. However, findings regarding combined degenerative variables have not been entirely consistent. Listi et al. reported that using only osteophyte variables made a more accurate model than using combined osteophyte variables and facet joint osteoarthritis. This suggests that adding facet parameters did not substantially improve the model performance in their group [11]. The present study showed better apparent performance for the multivariable models than for the univariable models within the development sample. In the combined sample, model 1 achieved an R^2^ of 0.84 with a RMSE of 6.93 years. Furthermore, unlike the findings of Listi et al. [11], the combined osteophyte–facet model (model 2) in our study also resulted in improved predictive performance (R^2^ = 0.75, RMSE = 8.58), indicating that the integration of additional degenerative parameters may provide additive value to adult age estimation. Details of previous studies and the predictive performance of the present model are presented in Table 8.

Performance of each model was further investigated by analyzing prediction accuracy and bias throughout decades. In accordance with prior literature [27], both models demonstrated relatively better performance for individuals in their 20s in the combined sample, however, the negative bias in this age group indicated systematic age overestimation. Prediction within ±10 years of chronological age was achieved in 84.3% of cases for model 1 and 75.0% for model 2. This finding was broadly comparable with earlier multivariable regression studies [27], although direct comparison is limited by differences in sample design, predictor structure, and validation strategy. Prediction errors tended to increase in the older age groups, especially for model 2, with the greatest underestimation observed among individuals aged 70–79 years. This pattern may indicate that thoracic degenerative features become less discriminative at advanced ages, possibly because severe changes are more common within older age ranges. However, the present data do not allow this explanation to be confirmed. The findings of the present study should therefore be interpreted in light of this age-related variation in prediction performance.

Although the models showed relatively low MAE values and bias at the different age groups, the mean width of the 95% prediction intervals were 28.01 years for model 1 and 34.38 years for model 2. These relatively wide intervals indicate substantial uncertainty around individual age estimates and reflect a broader challenge in adult age estimation, as considerable interindividual variability limits the certainty of individual estimates [28]. The models should therefore be interpreted as complementary tools for adult age estimation.

From a forensic anthropology perspective, the potential applicability of the models is likely varied according to case context. The present models were developed from clinical CT examinations of, and their applicability to postmortem CT, decomposed bodies, and skeletal remains requires further evaluation. Postmortem changes, including autolysis and putrefaction, may particularly affect paraspinal muscle attenuation and should therefore be considered when evaluating model 1. In cases with a short postmortem interval, model 1 may represent a candidate approach for future postmortem CT validation, whereas the model 2 may be more suitable for evaluation under conditions in which attenuation measurements are unreliable. In skeletal remains, vertebral preservation may be incomplete; therefore, region-specific vertebral approaches remain valuable [24]. In situations where only partial vertebral segments are available, osteophyte-related variables such as L-max may provide a basis for further validation as individual predictors [8].

Several limitations of this study should be acknowledged. First, the retrospective design limited the availability of detailed sociodemographic, clinical, and lifestyle-related data. Information on occupational status, ancestry, socioeconomic status, physical activity, smoking, and nutritional status was not systematically available and therefore could not be included as covariates in the study. These factors may influence skeletal degeneration and attenuation-related measurements, and their absence limits the biological interpretation of the observed associations. Second, CT examinations were performed using different scanner models and acquisition protocols. Variations in scanner characteristics and contrast administration may affect attenuation measurements, particularly paraspinal muscle attenuation. Quantitative CT protocols using calibration phantoms may support the standardization of measurement; however, the retrospective design of the present study did not allow the implementation of such approaches. Because the improved performance of model 1 depends partly on attenuation-based measurements, this model should be regarded as preliminary until its performance has been confirmed across different scanners and acquisition protocols. Third, the models were developed from CT scans of living individuals; their applicability to postmortem CT and decomposed bodies remains to be validated. Finally, the model was developed using data from a single centre. Although internal validation was performed, the resulting performance estimates do not replace validation in an independent cohort. External validation across different populations, institutions, scanner platforms, and acquisition protocols is therefore necessary to determine the reproducibility of the models. Until such validation is available, the models should be considered exploratory rather than operational tools for routine forensic age estimation.

Despite these limitations, the study has several strengths. It evaluates multiple morphological and attenuation-based parameters within a multivariable regression framework, provides two models that may be considered under different forensic data-availability conditions, and incorporates bootstrap internal validation to obtain optimism-corrected estimates of model performance.

## 5. Conclusions

This study presents population-specific, regression-based models for adult age estimation using thoracic MDCT scans. By combining morphologically graded degenerative variations with vertebral bone and paraspinal muscle attenuation measurements, the models provide a comprehensive assessment of age-related degeneration. When compared with osteophyte-based regression models reported in the literature, the present multivariable models demonstrated generally comparable. The inclusion of attenuation parameters was associated with lower prediction errors in the development sample, although the individual prediction intervals remained relatively wide.

The proposed dual-model strategy may have potential value for evaluating thoracic vertebral parameters under different forensic conditions. The non-restricted model may be considered when CT attenuation measurements of soft tissue are reliable, whereas the morphology-based model does not require attenuation data. However, neither model was directly evaluated in postmortem CT, decomposed bodies, or skeletal remains. The models should therefore be regarded as complementary and exploratory CT-based approaches for adult age estimation. External validation in multicenter and postmortem cohorts is needed to establish the generalizability of the models and assess their suitability for routine forensic application.

## Figures and Tables

**Table 1:** Measurements of parameters.

	Grades	Definitions
Osteophyte *		
Linear Classification	0	No formation
	1	<2 mm
	2	2–3.9 mm
	3	4–5.9 mm
	4	6–7.9 mm
	5	≥8 mm
Morphological Classification	0	No formation
	1	Slightly spur formation
	2	Spur which exceeds line between vertebrae
	3	Fusion/Bridging
**Facet †**		
	0	Normal
	1	Irregularity on the joint surface and/or small osteophytes
	2	Moderate osteophytes
	3	Large osteophytes and/or fusion of joint and/or subchondral cyst formation

Note: * Based on Chiba [8], † based on Weishaupt [19].

**Table 2:** Definitions and abbreviations of variables.

Parameters	Variables	Definitions
Osteophyte	L-sum	Sum of linear grade of each surface (superior and inferior margin) of thoracic vertebra
	L-max	Max linear grade of all thoracic column
	L0	Number of vertebral surfaces without osteophyte formation
	L2–5	Number of vertebra surface which has 2–5 linear grade
	M-sum	Sum of morphological grade of each surface of thoracic vertebra
	M 3	Number of vertebral surfaces with morphologic grade 3
Facet	F-sum	Sum of facet grades at thoracic vertebrae
	F0	Number of facets which has grade 0
	F2-3	Number of facets which has grade 2 or 3
Bone Attenuation	B-T4	Attenuation of vertebra corpus on T4
	B-T8	Attenuation of vertebra corpus on T8
	B-T12	Attenuation of vertebra corpus on T12
Muscle Attenuation	M-T4	Mean attenuation of paraspinal muscle on level T4
	M-T8	Mean attenuation of paraspinal muscle on level T8
	M-T12	Mean attenuation of paraspinal muscle on level T12

**Table 3:** Descriptive characteristics of the variables in study sample.

Variables	Female	Male	*p*-Value
L-sum	16 (9–29)	20.50 (7–38.5)	0.129
L-max	2 (1.5–3)	3 (2–5)	**0.009**
L0	12 (7.5–16)	10.50 (5.5–18)	0.454
L2–5	3.5 (0.5–9)	5 (1–13)	**0.043**
M-sum	14.5 (8–22)	16.50 (6.5–30.5)	0.155
M3	0 (0–2)	0 (0–4)	**0.006**
F-sum	4 (2–9)	4 (2–6)	0.421
F0	8 (5–10)	8 (6–10)	0.514
F2-3	0 (0–1)	0 (0–0)	**0.022**
B-T4 †	220.67 ± 64.98	227.80 ± 61.39	0.383
B-T8 †	201.57 ± 66.84	201.00 ± 62.71	0.946
B-T12 †	191.64 ± 67.93	186.87 ± 60.68	0.566
M-T4	23.5 (18–30.5)	24 (17–33)	0.705
M-T8	33.75 (25.75–41.5)	38.5 (23.5–46.25)	0.078
M-T12	41.75 (31.25–49.75)	46.5 (36.25–53.5)	**0.003**

Data are presented as median (interquartile range) unless otherwise indicated. Variables marked with † showed a normal distribution and are presented as mean ± standard deviation; these variables were compared using the independent-samples t-test, whereas all other variables were compared using the Mann–Whitney U test. Bold values indicate statistical significance (*p* < 0.05).

**Table 4:** Multivariable regression equations for age estimation.

Models	Equations
Combined	
Model 1	Age = 86.771 − 0.838(L0) − 0.078(B-T12) − 1.331(F0) − 0.160(M-T12) + 1.807(L-max)
Model 2	Age = 72.050 − 1.114(L0) − 1.982(F0) + 2.342(L-max)
Female	
Model 1	Age = 91.492 − 0.975(L0) − 0.085(B-T12) − 1.467(F0) − 0.148(M-T12) + 3.334(L-max) − 0.226(L-sum)
Model 2	Age = 77.426 − 1.079(L0) − 2.717(F0) + 5.322(L-max) − 0.336(L-sum)
Male	
Model 1	Age = 70.453 − 0.947(L0) − 0.073(B-T12) + 1.073(F-sum) + 1.691(L-max) − 0.132(M-T12)
Model 2	Age = 68.652 − 0.901(L0) − 1.904(F0) + 0.897(L2–5)

**Table 5:** Apparent and bootstrap optimism-corrected performance of multivariable linear regression models in the combined and sex-specific samples.

	Apparent (in Sample)			Bootstrap Optimism Correction		
	R^2^	RMSE	MAE	R^2^	RMSE	MAE
Combined						
Model 1	0.84	6.93	5.53	0.83	7.23	5.77
Model 2	0.75	8.58	6.91	0.74	8.83	7.11
Female						
Model 1	0.85	6.71	5.4	0.82	7.36	5.93
Model 2	0.76	8.48	7.04	0.74	9.01	7.48
Male						
Model 1	0.84	6.97	5.54	0.81	7.71	6.11
Model 2	0.78	8.06	6.68	0.76	8.59	7.11

Apparent performance was calculated in the development sample. Optimism-corrected estimates were obtained from 1000 bootstrap resamples, with the complete stepwise variable-selection procedure repeated within each resample. R^2^: coefficient of determination, RMSE: root mean squared error, MAE: mean absolute error.

**Table 6:** Absolute age estimation error distribution of age predictions (%).

Group	Model	0–5 Years	5–10 Years	10–15 Years	15–20 Years	>20 Years
Combined	Model 1	53.5	30.8	12.9	2.9	0
	Model 2	42.5	32.5	15	8.8	1.2
Female	Model 1	53.3	30.8	13.3	2.5	0
	Model 2	35	44.2	12.5	7.5	0.8
Male	Model 1	53.3	30.8	12.5	3.3	0
	Model 2	41.7	34.2	18.3	5.8	0

**Table 7:** Decade-specific mean absolute error and bias of the multivariable regression models.

		Total	20–29	30–39	40–49	50–59	60–69	70–79
Model 1-combined	MAE	5.53	4.9	5.52	5.56	5.61	5.95	5.67
	Bias	0	−3.55	−2.33	−1.46	1.02	2.28	4.04
Model 2-combined	MAE	6.91	4.85	7.73	6.53	6.76	7.27	8.32
	Bias	0	−3.39	−5.28	−3.72	1.16	4.69	6.54
Model 1- female	MAE	5.4	5.11	5.96	4.38	4.9	5.88	6.17
	Bias	0	−3.53	−2.7	−0.6	1.64	0.32	4.88
Model 2- female	MAE	7.04	5.74	7.89	6.2	6.51	7.1	8.81
	Bias	0	−3.25	−5.53	−3.42	1.64	3.5	7.06
Model 1-male	MAE	5.54	4.38	5.2	6.48	5.82	5.58	5.77
	Bias	0	−2.87	−1.8	−2.5	0.04	3.26	3.87
Model 2-male	MAE	6.68	4.62	6.44	7.25	6.86	7.64	7.29
	Bias	0	−3.63	−3.64	−3.3	0.73	4.87	4.97

MAE, mean absolute error. Bias was calculated as observed chronological age minus predicted age; positive values indicate underestimation, whereas negative values indicate overestimation.

**Table 8:** Overview of osteophyte-based adult age estimation using regression models in previous studies.

Study, Year	Technique, Obtaining	Population	Sample Size	Age (Years)	Variable	R^2^ *	Accuracy *
Snodgrass (2004) [21]	VA, Skeletal remains	North American	384	20–80	Osteophyte	0.49 in female, 0.53 in male	NR
Watanabe (2006) [9]	VA, Autopsy	Japanese	225	20–88	Osteophyte	0.713 in female, 0.730 in males	13.5 in female, 10.7 in male (SEE)
Listi (2012) [11]	VA, Skeletal remains	North American	104	30–90	Osteophyte, facet joints	0.408 (osteophyte), 0.319 ^†^	NR
Praneatpolgrang (2019) [24]	VA, Skeletal remains	Thai	400	22–97	Osteophyte	0.560 in combined sample	10.37 in combined sample (SEE)
Chiba (2022) [8]	PMCT, Cadavers	Japanese	250	20–95	Osteophyte	0.802 in females, 0.796 in males	9.55 in females, 9.88 in males (SEE)
Schanandore (2024) [27]	MDCT, Living individual	North American	319	10–89	Osteophyte	0.85 in combined sample ^†^	8.53 (RMSE)
Present Study	MDCT, Living individual	Turkish	240	20–79	Osteophyte, facet, bone and muscle attenuation	0.84 (AP) ^†^ 0.83 (after OC) ^†^ in combined sample	6.93 (RMSE, AP), 5.53 (MAE, AP), 7.23 (RMSE, OC), 5.77 (MAE, OC) in combined sample

* Values in the R^2^ column represent coefficient of determination, except for Ref. [9], which reported the correlation coefficient (r). Ref. [21] described the reported values as correlation coefficients (r^2^). Only the best-performing model from each referenced study is presented. ^†^ Multivariable regression model. AP: apparent performance, MAE: mean absolute error, MDCT: multidetector computed tomography, NR: Not reported in the original study, OC: bootstrap optimism-correction performance, PMCT: postmortem computed tomography, R^2^: coefficient of determination, RMSE: root mean squared error, SEE: standard error of estimate, VA: visual analysis.

## Data Availability

The datasets used and/or analyzed during the current study are available from the corresponding author upon reasonable request.

## Supplementary Materials

The following supporting information can be downloaded at: https://www.mdpi.com/article/10.3390/diagnostics16162528/s1, Table S1: Distribution of the study sample by age group and sex; Table S2: Intra- and inter-observer reliability; Table S3: Univariable linear regression results; Table S4: Distribition of absolute age-estimation errors based on out-of-bag predictions (%); Table S5: Decade-specific mean absolute error and bias based on out-of-bag predictions.

## Abbreviations

The following abbreviations are used in this manuscript:

HU	Hounsfield units
MAE	Mean absolute error
MDCT	Multidetector computed tomography
PMCT	Postmortem computed tomography
R^2^	Coefficient of determination
RMSE	Root mean squared error
SEE	Standard error of estimate
