# Supplementary material for: Multiparametric CT-Based Adult Age Estimation Using Thoracic Vertebral Degenerative Changes, Vertebral Bone and Paraspinal Muscle Attenuation: A Regression-Based Approach
Source: Diagnostics (Basel). 2026 Aug 11;16(16):2528. doi: 10.3390/diagnostics16162528 (PMC13511673; doi:10.3390/diagnostics16162528)
Supplement: Supplementary file 1 [file diagnostics-16-02528-s001.zip › diagnostics-4455013-supplementary.pdf]

Table S1. Distribution of the study sample by age group and sex.

Age groups	Female (n)	Male (n)
**20-29**	20	20
**30-39**	20	20
**40-49**	20	20
**50-59**	20	20
**60-69**	20	20
**70-79**	20	20
**Total**	120	120

Table S2. Intra- and inter-observer reliability.

Variables	Intra-observer ICC (95% Cl)	Inter-observer ICC (95% Cl)
L-sum	0.991 (0.982-0.996)	0.978 (0.939-0.991)
L-max	0.985 (0.968-0.993)	0.962 (0.917-0.982)
L0	0.984 (0.965-0.992)	0.925 (0.745-0.971)
L2-5	0.980 (0.957-0.990)	0.969 (0.931-0.986)
M-sum	0.986 (0.971-0.993)	0.979 (0.953-0.990)
M3	0.985 (0.969-0.993)	0.985 (0.970-0.993)
F-sum	0.975 (0.948-0.988)	0.854 (0.381-0.948)
F0	0.967 (0.931-0.984)	0.835 (0.270-0.943)
F2-3	0.943 (0.880-0.973)	0.860 (0.691-0.935)
B-T4	0.958 (0.912-0.980)	0.938 (0.870-0.970)
B-T8	0.929 (0.851-0.966)	0.909 (0.809-0.957)
B-T12	0.962 (0.919-0.982)	0.958 (0.908-0.981)
M-T4	0.925 (0.844-0.964)	0.910 (0.812-0.957)
M-T8	0.981 (0.961-0.991)	0.968 (0.933-0.985)
M-T12	0.979 (0.957-0.990)	0.965 (0.927-0.983)

ICC values are presented with 95% confidence intervals. ICC, intraclass correlation coefficient; CI, confidence interval.

Table S3. Univariable linear regression results.

Variables	Correlation coefficient	Regression equation	R^2	RMSE	MAE	p-value
**Combined**						
L-sum	0.828	y= 34.229 + 0.680x	0.567	11.37	9.44	<0.001
L-max	0.773	y= 26.504 + 8.873x	0.565	11.40	9.04	<0.001
L0	-0.822	y= 74.667 - 2.085x	0.682	9.75	7.55	<0.001
L2-5	0.813	y= 35.989 + 2.162x	0.578	11.23	9.20	<0.001
M-sum	0.820	y= 33.123 + 0.881x	0.576	11.25	9.26	<0.001
M3	0.629	y= 44.797 + 2.226x	0.261	14.85	12.57	<0.001
F-sum	0.748	y= 33.379 + 3.153x	0.506	12.14	10.05	<0.001
F0	-0.737	y= 83.830 – 4.485x	0.541	11.71	9.31	<0.001
F2-3	0.553	y= 44.979 + 7.807x	0.231	15.16	12.71	<0.001
B-T4	-0.602	y= 85.632 – 0.161x	0.341	14.02	11.64	<0.001
B-T8	-0.667	y= 83.709 – 0.170x	0.399	13.40	11.04	<0.001
B-T12	-0.740	y= 86.355 – 0.195x	0.519	11.99	9.83	<0.001
M-T4	-0.537	y= 66.554 – 0.655x	0.228	15.18	12.77	<0.001
M-T8	-0.560	y= 70.741 – 0.601x	0.269	14.77	12.24	<0.001
M-T12	-0.674	y= 83.830 – 0.804x	0.397	13.42	11.06	<0.001
**Female**						
L-sum	0.816	y= 34.322 + 0.762x	0.542	11.72	9.71	<0.001
L-max	0.787	y= 24.737 + 10.651x	0.587	11.13	9.09	<0.001
L0	-0.805	y= 76.231 – 2.172x	0.655	10.17	7.97	<0.001
L2-5	0.796	y= 37.408 + 2.257x	0.530	11.87	9.87	<0.001
M-sum	0.819	y= 33.344 + 0.961x	0.545	11.68	9.53	<0.001
M3	0.622	y= 45.825 + 2.397x	0.221	15.29	12.91	<0.001
F-sum	0.804	y= 32.500 + 3.096x	0.589	11.10	9.07	<0.001
F0	-0.817	y= 84.525 – 4.680x	0.655	10.17	8.14	<0.001
F2-3	0.624	y= 43.524 + 8.148x	0.302	14.47	12.22	<0.001
B-T4	-0.629	y= 86.668 – 0.168x	0.396	13.46	11.10	<0.001
B-T8	-0.707	y= 86.578 – 0.184x	0.500	12.25	9.92	<0.001
B-T12	-0.768	y= 87.162 – 0.197x	0.589	11.10	8.96	<0.001
M-T4	-0.545	y= 66.258 – 0.648x	0.235	15.15	12.75	<0.001
M-T8	-0.566	y= 75.204 – 0.765x	0.325	14.23	11.59	<0.001
M-T12	-0.672	y= 85.044 – 0.888x	0.432	13.05	10.73	<0.001
**Male**						
L-sum	0.846	y = 33.313 + 0.649x	0.623	10.64	8.91	<0.001
L-max	0.782	y= 25.772 + 8.301x	0.602	10.92	8.64	<0.001
L0	-0.842	y= 73.410 – 2.021x	0.714	9.26	7.13	<0.001
L2-5	0.844	y= 33.979 + 2.173x	0.655	10.18	8.29	<0.001
M-sum	0.832	y= 32.230 + 0.849x	0.631	10.52	8.71	<0.001
M3	0.661	y= 43.572 + 2.202x	0.320	14.28	12.03	<0.001
F-sum	0.684	y= 33.793 + 3.318x	0.438	12.98	10.91	<0.001
F0	-0.653	y= 82.969 – 4.277x	0.437	13.0	10.46	<0.001
F2-3	0.481	y= 46.227 + 7.701x	0.177	15.71	13.15	<0.001
B-T4	-0.544	y= 84.594 – 0.154x	0.296	14.53	12.21	<0.001
B-T8	-0.546	y= 80.467 – 0.154x	0.309	14.40	12.14	<0.001
B-T12	-0.674	y= 85.586 – 0.193x	0.454	12.80	10.67	<0.001

M-T4	-0.524	y= 66.872 – 0.663x	0.229	15.21	12.77	<0.001
M-T8	-0.550	y= 68.615 – 0.516x	0.248	15.02	12.57	<0.001
M-T12	-0.699	y= 85.428 – 0.792x	0.401	13.40	11.10	<0.001

Correlation coefficient (Spearman's rho) values indicate age-variable correlations, whereas R^2 values were derived from univariable linear regression models and therefore are not expected to correspond to the squared rho values. R^2: coefficient of determination, RMSE: root mean squared error, MAE: mean absolute error.

Table S4. Distribution of absolute age-estimation errors based on out-of-bag predictions (%).

Group	Model	0-5 years	5-10 years	10-15 years	15-20 years	>20 years
**Combined**	Model 1	52.5	30.4	12.9	4.2	0
	Model 2	41.7	31.2	17.1	8.3	1.7
**Female**	Model 1	50	33.3	11.7	5	0
	Model 2	31.7	42.5	17.5	6.7	1.7
**Male**	Model 1	52.5	28.3	11.7	6.7	0.8
	Model 2	41.7	30	20	8.3	0

Values are presented as percentages of cases within each absolute error interval. For each individual, the out-of-bag prediction was calculated as the mean of the predictions obtained from bootstrap iterations in which that individual was not included in the resampled development dataset.

Table S5. Decade-specific mean absolute error and bias based on out-of-bag predictions.

		Total	20-29	30-39	40-49	50-59	60-69	70-79
**Model 1-combined**	MAE	5.73	5.01	5.69	5.82	5.92	6.05	5.85
	Bias	0.04	-3.56	-2.37	-1.5	1.13	2.35	4.21
**Model 2-combined**	MAE	7.05	4.85	7.66	6.69	7.1	7.59	8.43
	Bias	-0.01	-3.64	-5.25	-3.7	1.2	4.73	6.61
**Model 1-female**	MAE	6	5.89	6.35	4.71	5.42	6.77	6.89
	Bias	0.03	-4.49	-2.57	-0.33	1.96	0.41	5.18
**Model 2-female**	MAE	7.44	5.73	7.89	6.34	7.36	8.21	9.13
	Bias	-0.06	-3.9	-5.66	-3.19	1.87	3.71	6.79
**Model 1-male**	MAE	5.86	4.41	5.39	7.1	6.28	6.06	5.96
	Bias	0.08	-3.11	-1.78	-2.73	0.6	3.63	3.83
**Model 2-male**	MAE	6.95	4.56	6.94	7.54	7.05	7.29	8.29
	Bias	-0.07	-3.53	-4.04	-3.71	0.43	4.83	5.59

For each individual, the out-of-bag prediction was calculated as the mean of the predictions obtained from bootstrap iterations in which that individual was not included in the resampled development dataset. MAE, mean absolute error. Bias was calculated as observed chronological age minus predicted age; positive values indicate underestimation, whereas negative values indicate overestimation.
